# Preclinical findings reveal the pharmacological targets of ferulic acid in the treatment of traumatic brain injury

**DOI:** 10.1002/fsn3.3036

**Published:** 2022-08-26

**Authors:** Qinghua Dong, Shenglin Yang, Huafeng Liao, Qi He, Junxin Xiao

**Affiliations:** ^1^ Intensive Care Unit Guilin Municipal Hospital of Traditional Chinese Medicine Guilin Guangxi People's Republic of China

**Keywords:** biotargets, ferulic acid, findings, network pharmacology, traumatic brain injury

## Abstract

Traumatic brain injury (TBI) is characterized by cellular damage and inflammation in lesioned brain tissue. Ferulic acid has been shown to have a melioration effect on neurological functions. However, the active pharmacological effects and the underlying mechanisms of ferulic acid against TBI remain unclear. On the basis of network pharmacology and molecular docking methodology, this study aimed to investigate the beneficial effects of ferulic acid in treating TBI, and characterized the detailed biotargets and mechanisms of these actions. The identified core targets were validated via in silico simulation. We identified 91 overlapping targets associated with ferulic acid and TBI. In‐silico simulation analysis validated the putative core targets of tumor protein p53, mitogen‐activated protein kinase (MAPK) 1, and estrogen receptor 1. The Gene Ontology‐enriched annotations and findings were largely associated with cell proliferation, apoptosis, and inflammation in nerve cells. Additional Kyoto Encyclopedia of Genes and Genomes enrichment analysis unmasked the pharmacological pathways of ferulic acid in treating TBI, including the MAPK signaling pathway and hypoxia‐inducible factor‐1 signaling pathway. Bioinformatic analyses and findings provide a new preclinical strategy for revealing the core targets and network pathways of ferulic acid in treating TBI. Moreover, some bioinformatic findings were computationally validated in silico for exhibiting the neuroprotective action of ferulic acid against TBI.

## Graphical abstract

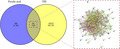

## 1 INTRODUCTION

Traumatic brain injury (TBI), which is a common cerebral trauma, refers to a pathological condition wherein brain lesions form because of mechanical trauma (Michinaga & Koyama, 2021). TBI can occur after common situations, including a traffic accident, unintentional falls, and a heavy hit to the head (Jodoin et al., 2016). As reported in China, the population of people with TBI is large, and a standardized pattern is absent (Gao et al., 2020). The preliminary onset of TBI is pathologically implicated in inflammatory outbreak and infiltration and is accompanied with lesioned neurocytes (Jodoin et al., 2016). In clinical valuation, the Glasgow Coma Scale is used to determine the severity of TBI and to establish a timing window for improving therapeutic efficacy (Palepu et al., 2021). In clinical prescription, hormonotherapy with estrogen and androgen is commonly used in TBI for axonal regeneration and neuroprotection (Acaz‐Fonseca et al., 2016; Martin‐Jiménez et al., 2019). Although hormonotherapy has a visible effect against TBI, a long period of uptake with these hormones may increase the risk of tumorigenesis in other tissues (Del Río et al., 2020). Therefore, the imperative task is to screen a candidate compound with pharmacological bioactivity than can effectively treat TBI. Traditional Chinese medicine has been historically used for TBI treatment because of its multitarget characteristics and effectiveness (Xia et al., 2020). Ferulic acid, which is separated from the botanic resin in *Ferula sinkiangensis* K. M. Shen, has antiradiation (Shao et al., 2018), antioxidant (Mamdouh et al., 2021), and antiviral (Ma et al., 2020) properties. An interesting report *in vivo* shows that ferulic acid may be used for TBI mitigation via the inhibition of neuronal apoptosis and oxidative stress (Erbil et al., 2019). Other existing evidence has not been used to reveal the anti‐TBI mechanisms of ferulic acid intervention. Therefore, any potential tool for uncovering ferulic acid‐ediated biological mechanism against TBI is needed. Network pharmacology has been recently conducted to identify bioactive compounds, disorders characterized by integrated mechanisms, and key target genes (Li, Li, et al., 2021; Oh et al., 2021). This strategy can prospectively assist in improving the use of natural ingredients in drug development for TBI treatment. In the current study, we aimed to apply an integrated method to identify the core targets and mechanisms of ferulic acid in TBI treatment on the basis of network pharmacology and molecular docking. Collectively, current bioinformatic studies can provide new insights into the therapeutic perspective regarding the treatment of TBI by ferulic acid.

## 2 MATERIALS AND METHODS

### 2.1 Screening ferulic acid's and TBI's targets

The databases of Traditional Chinese Medicine Systems Pharmacology Database and Analysis Platform (TCMSP), SwissTargetPrediction, PharmMapper, Bioinformatics Analysis Tool for Molecular mechANism (BATMAN), DrugBank, and SuperPred were used for the prediction of the candidate targets of ferulic acid. By using the databases of Genecard and DisGeNET, the targets of TBI were screened (Liu et al., 2016; Yang et al., 2022). Preliminary data were processed to eliminate duplicates by using the Uniprot tool before the final gene IDs were identified. The identified genes that overlap with ferulic acid and TBI were represented in a Venn diagram by using bioinformatics (http://www.uniprot.org/).

### 2.2 Establishing a protein–protein interaction (PPI) network and identifying core targets

The overlapping genes of ferulic acid and TBI were imported to Cytoscape 3.7.2 to construct a PPI network. The string tool was used for exhibiting PPIs, and the minimum interaction value was set to 0.09. In addition, the “Analyze network” plugin in Cytoscape was used to determine the parameters in protein interaction, including betweenness, degree of interactions, and closeness. The identified hubs in the network would be recognized as the core targets of ferulic acid against TBI (Killcoyne et al., 2009; Li, Wu, et al., 2021).

### 2.3 Gene ontology (GO) and pathway enrichment assay

The functional analysis of GO and Kyoto Encyclopedia of Genes and Genomes (KEGG) was performed using the Database for Annotation, Visualization, and Integrated Discovery bioinformatic resource tool to reveal the detailed pharmacological mechanisms of ferulic acid against TBI (Qin et al., 2021; Zhang & Zhang, 2022). Other bar and bubble charts from GO and KEGG were exhibited using R 3.6.1. Moreover, an overview of the data in all bioinformatic findings was visualized accordingly.

### 2.4 In‐silico molecular docking simulation

To verify the core targets in protein levels, molecular docking technology was used to better characterize the interaction activities between ferulic acid and TBI‐related core proteins. Regarding in silico procedures, the protein molecular structure of core targets was downloaded from the Protein Data Bank database (Zardecki et al., 2022). Chem Bio Office 2010 was used for 3D structure optimization with ChemBio3D Draw. Data were verified using Autodock Vina software. For binding assessment, the molecular docking setting was configured. To characterize the biological conformations in respective poses, the docking active center was scored for the binding energy between ligands and proteins. The optimal binding poses in every molecule were determined accordingly, and the molecular certification of the binding poses was assayed via Autodock Vina software by using a grid box setting (Forli et al., 2016). The docked parameters were scored and detailed for in silico visualization (Forli et al., 2016; Zardecki et al., 2022).

## 3 RESULTS

### 3.1 Definition of molecular targets in ferulic acid and TBI

**FIGURE 1:**
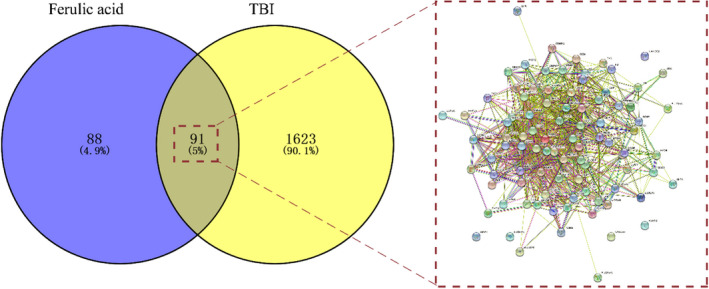
Additional 91 potential shared targets between ferulic acid and TBI in a Venn diagram characterized by gene–gene interactions in a global view.

To screen ferulic acid targets, 179 ferulic acid‐elated common genes were identified after eliminating duplicates. Additionally, the other disease genes of TBI were collected via database analysis. As shown in a Venn diagram, both ferulic acid and TBI have 91 overlapping targets (5%), and these shared genes were further analyzed for characterization with correlative nodes and edges (Figure 1).

### 3.2 PPI network and core targets in ferulic acid against TBI

**FIGURE 2:**
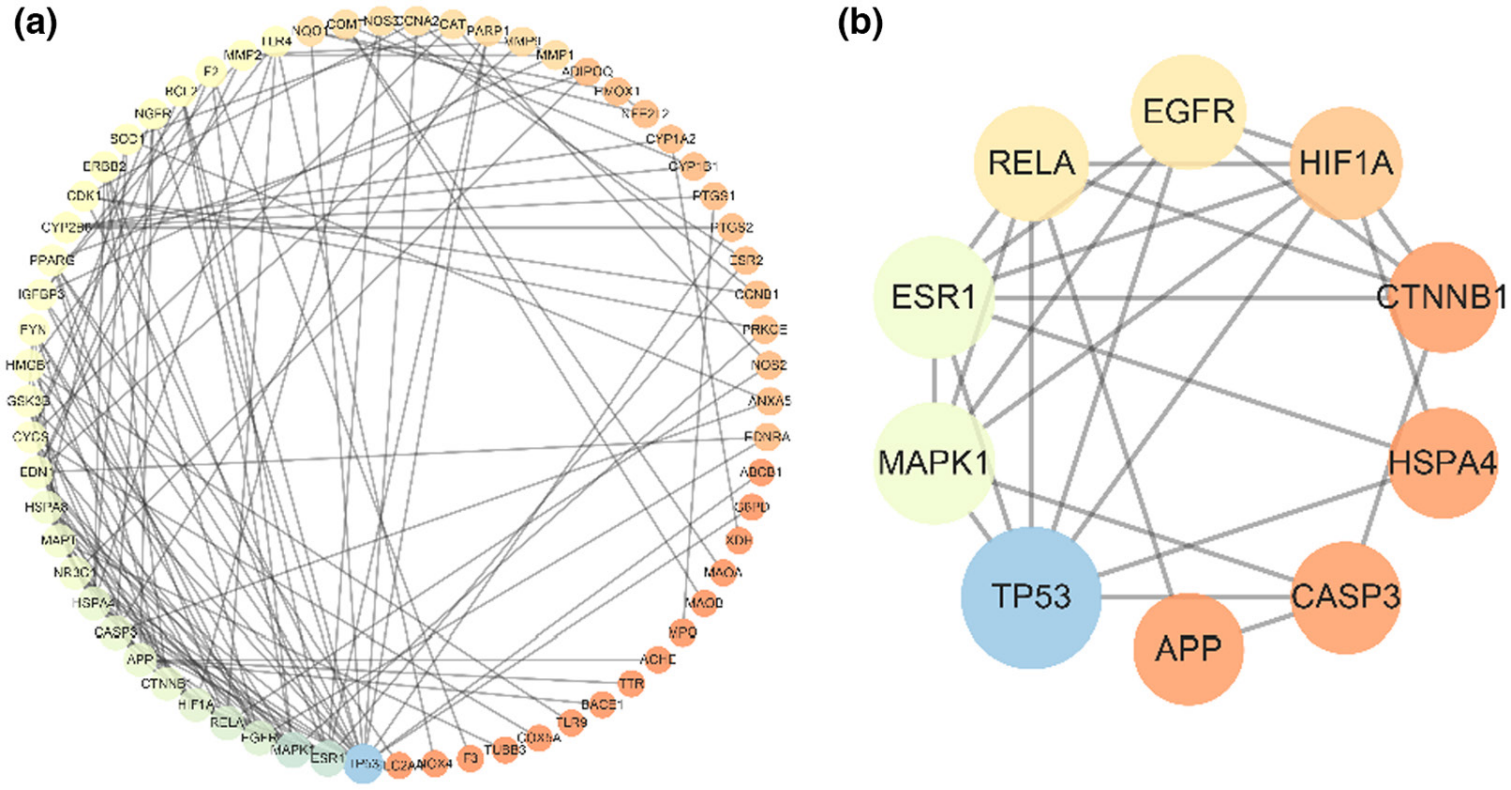
Interaction of a PPI network in overlapping targets. (a) Visualized layout in a PPI network in a degree‐scored circle. (b) Identification of all interaction core targets.

To identify the core targets in ferulic acid against TBI, we established a PPI network before revealing the therapeutic molecular mechanisms in TBI. All 91 target genes were integrated in a visualized PPI network, and these connecting targets with variant colorations indicated a scoring interaction (Figure 2a). Further analytical results suggested that the 10 core targets of *TP53, MAPK1, ESR1, EGFR, RELA, HIF1A, APP, HSPA4, CASP3*, and *CTNNB1* were identified from PPI network genes (Figure 2b).

### 3.3 Enrichment determination findings

**FIGURE 3:**
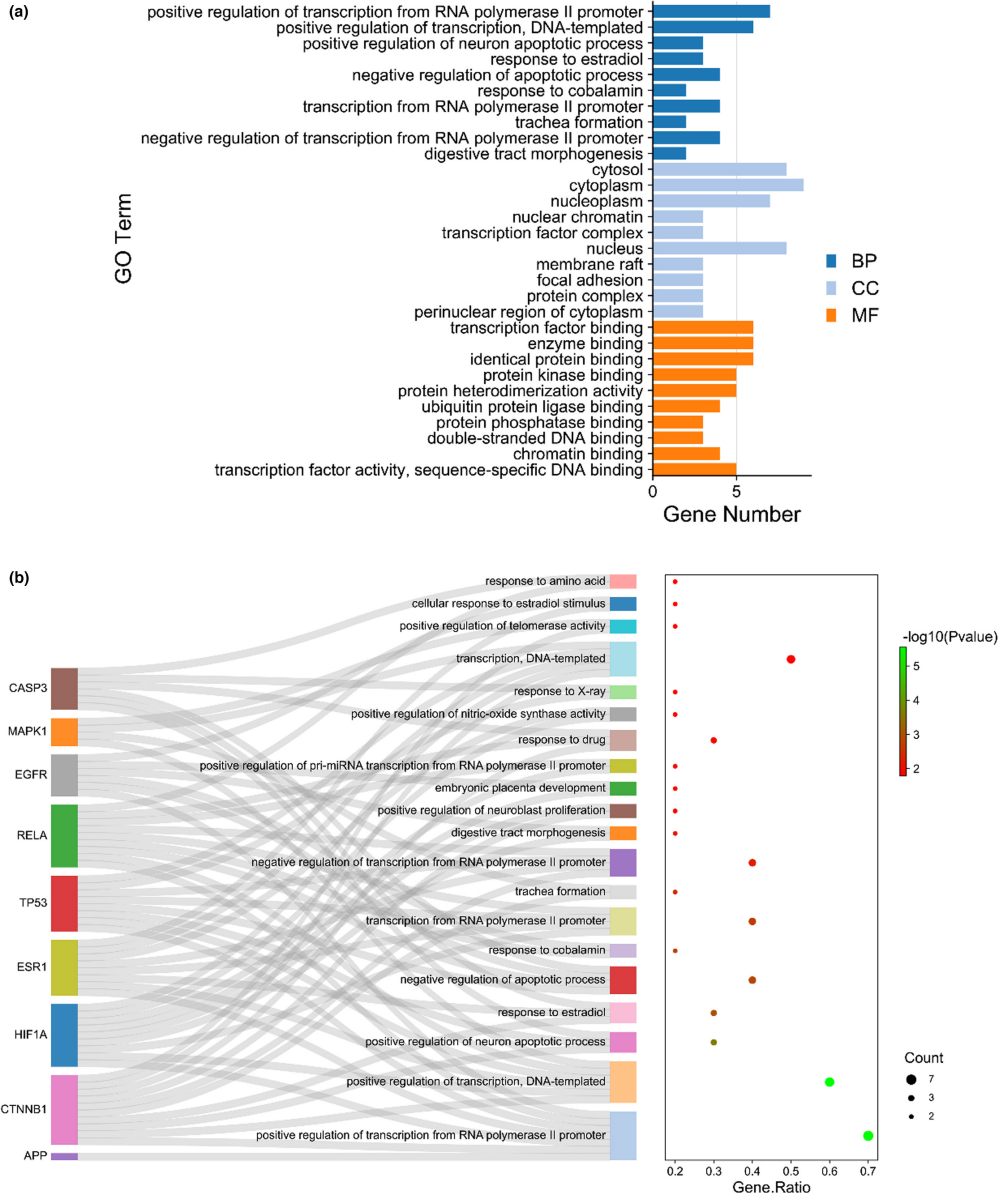
GO enrichment and functional analysis, including BPs, CCs, and MFs. The length in the histogram denotes the degree value in the core genes.

**FIGURE 4:**
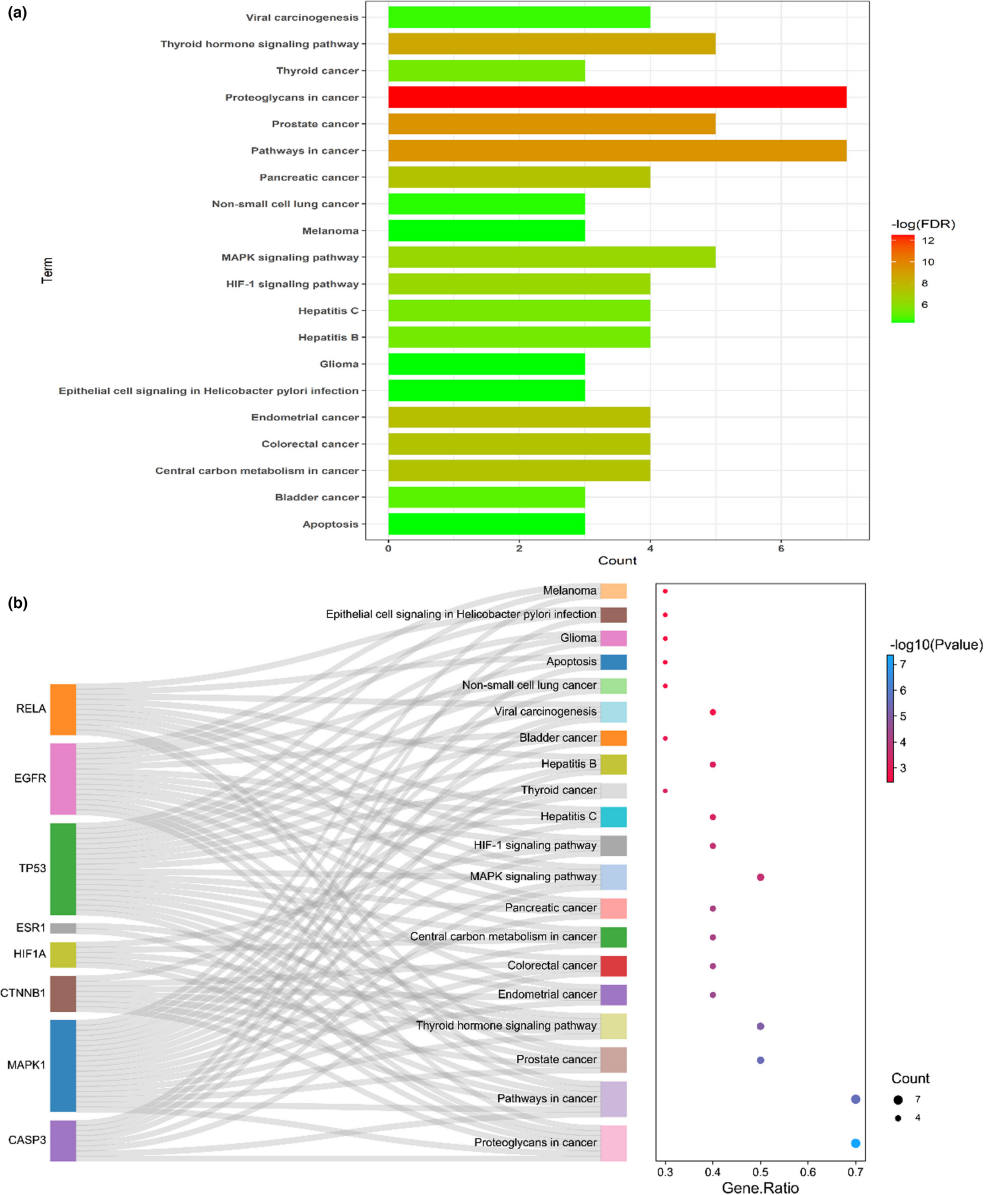
KEGG signaling pathway of ferulic acid–TBI targets and diagram of potent pharmacological pathways.

Detailed biological processes (BPs) were revealed using all core targets, including cellular compartments (CCs) and molecular functions (MFs), and top GO terms were screened and characterized accordingly (Figure 3). Among these top functional categories, the target genes in ferulic acid against TBI were actively involved in the BPs of the positive regulation of transcription from RNA polymerase II promoter, positive regulation of transcription (DNA templated), positive regulation of neuron apoptotic process, response to estradiol, negative regulation of apoptotic process, response to cobalamin, transcription from RNA polymerase II promoter, formation of the trachea, negative regulation of transcription from RNA polymerase II promoter, morphogenesis of the digestive tract, positive regulation of neuroblast proliferation, development of embryonic placenta, positive regulation of pri‐miRNA transcription from RNA polymerase II promoter, response to drugs, positive regulation of nitric oxide synthase activity, response to x‐rays, transcription (DNA templated), positive regulation of telomerase activity, cellular response to estradiol stimulus, and response to amino acids. CC‐related categories mainly involved the cytosol, cytoplasm, nucleoplasm, nuclear chromatin, transcription factor complex, nucleus, membrane raft, focal adhesion, protein complex, perinuclear region of the cytoplasm, apical part of the cell, receptor complex, Golgi apparatus, cell–cell junction, basolateral plasma membrane, and synapse. MF‐based findings were chiefly accompanied with transcription factor binding, enzyme binding, identical protein binding, protein kinase binding, protein heterodimerization activity, ubiquitin protein ligase binding, protein phosphatase binding, double‐stranded DNA binding, chromatin binding, transcription factor activity, sequence‐specific DNA binding, protein binding, nitric oxide synthase regulator activity, transcription regulatory region DNA binding, transcriptional activator activity, RNA polymerase II core promoter proximal region sequence‐specific binding, DNA binding, nuclear hormone receptor binding, histone acetyltransferase binding, repressing transcription factor binding, core promoter sequence‐specific DNA binding, and RNA polymerase II transcription factor binding. As revealed in the KEGG findings (Figure 4), 20 top signaling pathways were characterized, including proteoglycans in cancer, pathways in cancer, prostate cancer, thyroid hormone signaling pathway, endometrial cancer, colorectal cancer, central carbon metabolism in cancer, pancreatic cancer, MAPK signaling pathway, HIF‐1 signaling pathway, hepatitis C, thyroid cancer, hepatitis B, bladder cancer, viral carcinogenesis, non‐small cell lung cancer, apoptosis, glioma, epithelial cell signaling in *Helicobacter pylori* infection, and melanoma.

### 3.4 Combined bioinformatic findings

**FIGURE 5:**
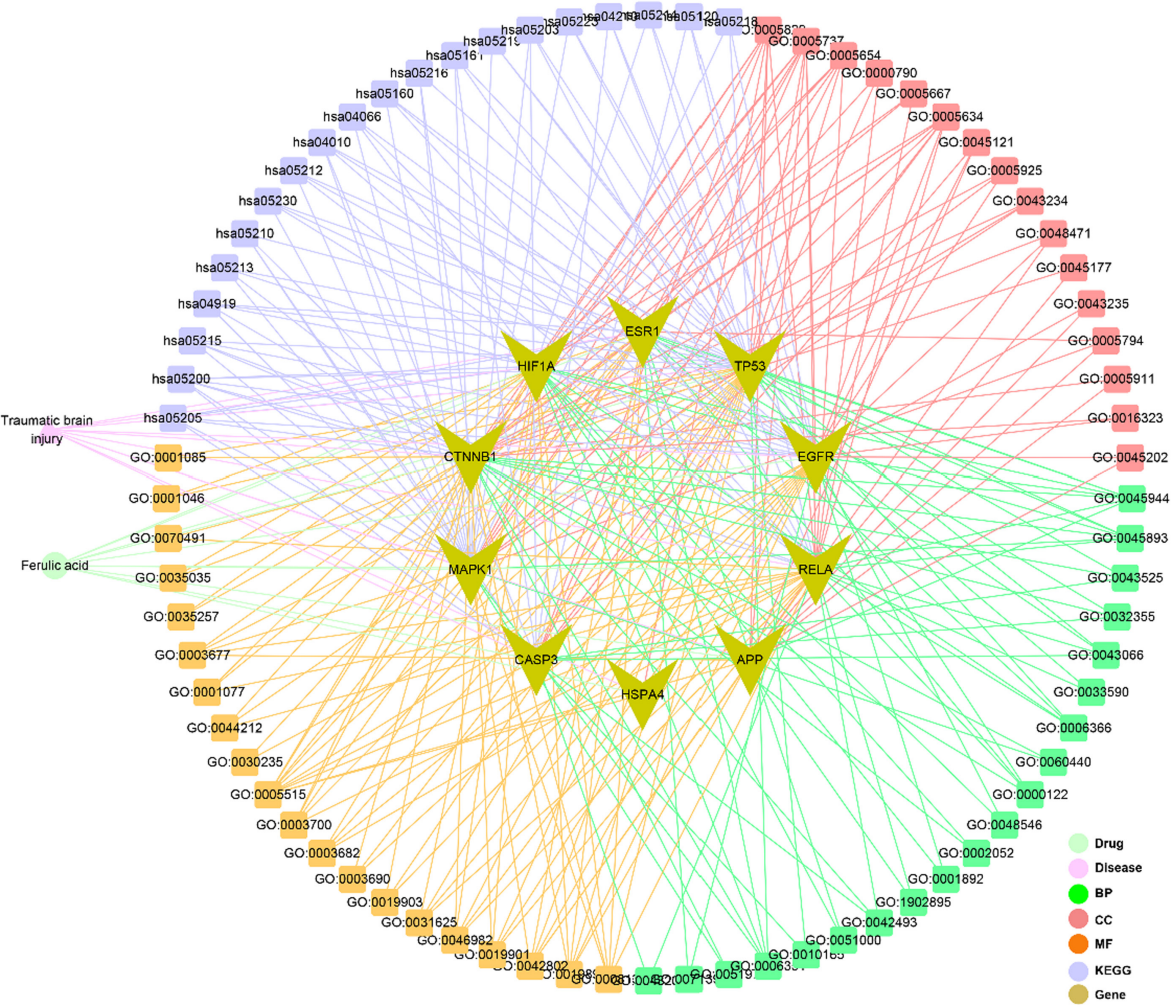
Outline of the integrated strategy using all bioinformatic data

The integrated network diagram was constructed for the visible presentation of TBI treatment via ferulic acid by using all‐core targets and enrichment data (Figure 5).

### 3.5 Molecular docking simulation data

**FIGURE 6:**
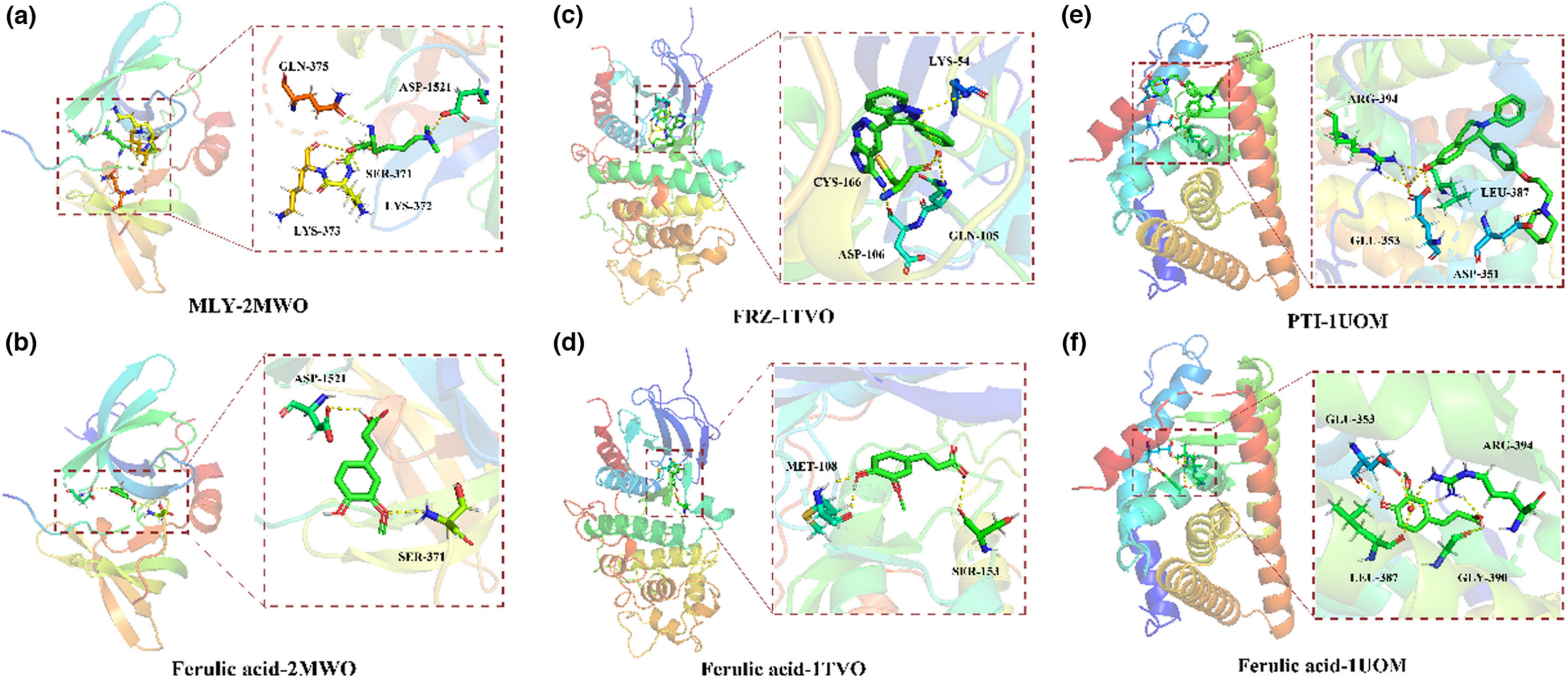
Molecular docking simulation of ferulic acid and human TP53, MAPK1, and ESR1. Three‐dimensional visualization of ferulic acid binding with (a,b) 2MWO in TP53, (c,d) 1TVO in MAPK1, and (e,f) 1UOM in ESR1.

We investigated the functional hypothesis that ferulic acid aimed to interact with key proteins in TBI, including TP53, MAPK1, and ESR1. In silico analysis suggested that ferulic acid showed a high binding energy to 2MWO in TP53 and interacted with residues of SER‐371 (2.5 Å) and ASP‐1521 (2.3 Å) (Figure 6a,b). As revealed in MAPK1, the in silico data showed that ferulic acid exhibited high binding energy to 1TVO and interacted with residues of SER‐153 (2.5 Å) and MET‐108 (2.1 Å) (Figure 6c,d). Additionally, ferulic acid displayed high binding energy to 1UOM in ESR1 and interacted with residues of LEU‐387 (2.9 Å) and ARG‐394 (2.7 Å) (Figure 6e,f).

## 4 DISCUSSION

To identify the pharmacological targets against TBI following ferulic acid treatment, all core targets and mechanisms of ferulic acid against TBI were identified on the basis of pharmacology network methods. Thus, we identified the core therapeutic genes/proteins that may act on candidate druggable targets via molecular docking simulation. Enrichment analysis revealed that the anti‐TBI pharmacological activities of ferulic acid are associated with the regulation of neuronal apoptosis, cell proliferation, and cerebral microenvironment. Ferulic acid is preclinically found in vivo with neuroprotective action, as shown in animal reports (Wang et al., 2021). Other pieces of evidence proved that TBI can trigger penetrating lesions in astroglia and microglia and cause inflammatory development (Milleville et al., 2020). Current GO findings indicated that some of the top BP categories was involved in the effective modulation of neuronal apoptosis. These bioinformatic data revealed the negative association between ferulic acid and TBI‐induced cell proliferation, thus implying that the inhibition of glial proliferation is one of the evident pharmacological activities of ferulic acid against TBI. Neuroinflammation is a driver for traumatic exacerbation via cascade inflammatory infiltration and cellular lesion (Delage et al., 2021). Increasing reports have proven that the antioxidation and antiinflammatory properties of ferulic acid is well recognized in preclinical studies (Shi et al., 2021). Current findings demonstrated that an antineuroinflammation action may be one of the pharmacological mechanisms of ferulic acid in treating TBI. Characterizing the pathogenesis associated with TBI may be vital for screening potent bioactive ingredients because current pharmacotherapy is lacking in the treatment of patients with TBI. By using network pharmacology and molecular docking approaches, the current study identified some of the core genes/proteins involved in the neuroprotective effects of ferulic acid in TBI. TP53 has been identified as having a pivotal role in apoptosis (Kastenhuber & Lowe, 2017). Some clinical findings indicate that p53 exerts a potent role in functional recovery in TBI, thus suggesting that a p53 treatment strategy can be used in TBI (Mellett et al., 2020). MAPKs, including MAPK1, is a well‐reported kinase for diversified biological functions, such as inflammation, apoptosis, cancerization (Kaur & Goyal, 2021). Targeting the suppression of MAPK1 may be a potential treatment for TBI‐induced neuronal damage (Bhowmick et al., 2019). ESR1, which is a transcription factor, may play a neuroregulation role (Lambert et al., 2018). Other preclinical data underline that ESR1 agonists may meliorate neurological function in TBI rats, thus indicating the possible neuroprotective action of ESR1 agonist (Khaksari et al., 2021). The in‐silico analysis in the current study revealed that TBI treatment using ferulic acid is associated with PPIs in BPs, including TP53, MAPK1, and ESR1. The neuroprotective effects of ferulic acid in treating TBI might be accomplished via synergetic interactions with apoptosis‐ and inflammation‐related signaling pathways. However, current bioinformatic discoveries should be included in further research validation, and additional experiments are still needed.

## 5 CONCLUSIONS

In summary, we applied emerging and integrated tactics to reveal the core targets and mechanisms of ferulic acid in the treatment of TBI by using the network pharmacology approach. Some of the core targets/proteins were further verified using molecular docking simulation. This study offers bioinformatic data‐based theoretics for the in‐depth revelation of anti‐TBI mechanisms mediated by ferulic acid, and provides an underlying foundation for future clinical applications.

## CONFLICT OF INTEREST

None.
